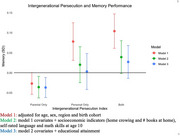# Intergenerational Discrimination, Dispossession, and Political Persecution: Associations with Memory in Older Adults from Central and Eastern Europe

**DOI:** 10.1002/alz70860_106803

**Published:** 2025-12-23

**Authors:** Brittany Koch‐Hale, Martin Fischer, Katrin Wolfova, Justina F. Avila, Matěj Kučera, Pavla Brennan Kearns, Dominika Seblova

**Affiliations:** ^1^ Second Faculty of Medicine Charles University, Prague, Czech Republic; ^2^ Faculty of Medicine Lund University, Lund, Sweden; ^3^ Columbia University, New York, NY, USA; ^4^ Columbia University Irving Medical Center, New York, NY, USA; ^5^ Vrije Universiteit Amsterdam, Amsterdam, Netherlands; ^6^ The National Institute of Mental Health, Klecany, Czech Republic

## Abstract

**Background:**

Discrimination and persecution can have lasting effects on socioeconomic status (SES) and health, including cognitive function. Central and Eastern Europe (CEE) has a history of war, political repression, and systemic discrimination, contributing to historical adversity across generations. However, the long‐term effects of these experiences on memory performance in older adults remain largely unexplored.

**Aim:**

To examine the association between an intergenerational index of discrimination, persecution, and dispossession and memory performance in older adults from CEE countries.

**Methods:**

Respondents from 11 CEE countries were interviewed in wave 7 of the Survey of Health, Ageing, and Retirement in Europe (*n* = 21,989; 59% female; 67.1 mean age). We constructed a categorical index for intergenerational experiences of discrimination, persecution, and dispossession: (0) no personal or parental exposure, (1) parental experience only (e.g., forced displacement, labor camp, or injury), (2) personal experience only (e.g., job‐related consequences of discrimination, or property dispossession), and (3) both personal and parental exposure. The memory performance z‐score was created by standardizing and combining immediate and delayed recall scores. First linear regression model adjusted for age, sex, CEE region, birth cohort and included country‐fixed effects. Second model additionally included self‐reported math and language abilities at age 10, childhood SES, and educational attainment; potential confounders depending on the timing of discrimination.

**Result:**

36.7% had some experience of discrimination or persecution, with 23.2% reporting parental exposure, 6% reporting only personal exposure and 7.5% reporting both. Respondents whose parents experienced discrimination or persecution had ‐0.03 lower memory scores (95%CI: ‐0.05; 0.00), whereas those reporting personal or both experiences had higher memory scores (β=0.08 95%CI: 0.03;0.12 & β=0.10 95%CI 0.06; 0.15). However, after adjusting for early life factors and educational attainment only the negative association between parental experiences of persecution and memory persisted (β=‐0.04, 95%CI: ‐0.06; ‐0.01).

**Conclusion:**

Having parents who were exposed to discrimination or persecution was associated with lower memory performance in older age. These findings suggest that adversity may have a lasting negative impact on cognitive function across generations.

Support: PRIMUS research program (PRIMUS/22/MED; PI Seblova)